# Probiotics Can Further Reduce Waist Circumference in Adults with Morbid Obesity after Bariatric Surgery: A Systematic Review and Meta-Analysis of Randomized Controlled Trials

**DOI:** 10.1155/2021/5542626

**Published:** 2021-04-01

**Authors:** Yu Zhang, Tong Yan, Chenxin Xu, Huawu Yang, Tongtong Zhang, Yanjun Liu

**Affiliations:** ^**1**^ The Affiliated Hospital of Southwest Jiaotong University, Chengdu, China; ^**2**^ Department of Endocrinology and Metabolism, Affiliated Hospital of Southwest Jiaotong University, The Third People's Hospital of Chengdu, Chengdu, China; ^**3**^ The Center of Gastrointestinal and Minimally Invasive Surgery, Affiliated Hospital of Southwest Jiaotong University, The Third People's Hospital of Chengdu, Chengdu, China; ^**4**^ Medical Research Center, Affiliated Hospital of Southwest Jiaotong University, The Third People's Hospital of Chengdu, Chengdu, China

## Abstract

Whether probiotics could be used as an adjunct to bariatric surgery is controversial. This meta-analysis aimed to evaluate the effects of probiotics on body weight, body mass index (BMI), percentage of the excess weight loss (%EWL), waist circumference (WC), and C-reactive protein (CRP) in adults with obesity after bariatric surgery (BS). PUBMED, EMBASE, and the Cochrane Central Registry of Controlled Trials were searched from the earliest record to March 2020. All randomized controlled trials (RCTs) on the effects of probiotics in adults with obesity after bariatric surgery were analyzed according to the eligibility criteria. Four RCTs, including 172 participants, were analyzed. There was a statistically significant difference in probiotics in the reduction of waist circumference at 12 months after bariatric surgery. However, probiotics were not effective in weight, BMI, %EWL, WC, and CRP both within 3 months and at 12 months postoperation. Probiotics aid adults with morbid obesity in achieving further waist circumference improvement after BS, with no significant effect on weight, BMI, %EWL, and CRP. More quality clinical studies are needed to confirm the efficacy and safety of probiotics, and address a number of practical issues before the routine clinical use of probiotics in adults with obesity undergoing BS.

## 1. Introduction

Obesity is on the rise globally and multiplies the risk of metabolic diseases [1]. Bariatric surgery is an effective way to treat obesity and metabolic diseases, and one mechanism is to change the gut microbiome [2–4]. However, the beneficial changes in gut microbiota do not seem to be sustainable. The improvement of gut microbial gene richness following BS was showed to be partially restored in most patients, showed recently [5]. Moreover, deficiencies in micronutrients are common, while other disorders have also been reported, such as small intestinal bacterial overgrowth, weight regain, and abdominal symptoms [6–8]. These mean that additional strategies should be involved to improve further gut microbiota and clinical outcomes after BS.

The reversal effects on the host's obesity and metabolism issues promoted by modulators of intestinal microbiota, such as probiotics, have been successfully demonstrated. Probiotics were observed in animals to prevent and combat weight gain and improve obesity-related metabolic disorders, such as insulin resistance, disturbance of lipid metabolism, and reduction in systemic inflammation [9–12]. Some other evidence supports the potential modulating effects that probiotic supplements can reduce body weight and visceral fat mass [13, 14], as well as metabolic parameters in humans [15–17].

The clinical value of probiotic supplements as an adjunctive therapy among subjects with obesity undergoing BS was inconsistent. This systematic review and meta-analysis of randomized clinical trials aim to evaluate the adjuvant effect of probiotics on weight loss in patients with obesity after BS.

## 2. Materials and Methods

This systematic review and meta-analysis were prepared under the Preferred Reporting Items for Systematic Reviews and Meta-Analyses (PRISMA) guidelines [18].

### 2.1. Eligibility Criteria

Here are the inclusion criteria. (1) The study design was a randomized controlled clinical trial (RCT). (2) The participants were adults (≥18 years) with obesity (body mass index ≥30 kg/m2) and received any kind of bariatric surgeries. They also had short-term or long-term follow-up. (3) The study compared any type of probiotics or synbiotics (a combination of probiotics and prebiotics) with placebo [19]. (4) The study should have shown at least one of the following items: weight, body mass index (BMI), percentage of the excess weight loss (%EWL), waist circumference (WC), and inflammatory factors.

Studies including patients who had undergone any other gastrointestinal surgeries were excluded. Also excluded were studies comparing probiotics with other interventions instead of placebo, as they also affect the outcomes.

### 2.2. Search Strategy

Two independent researchers searched the electronic databases of PUBMED, EMBASE, and the Cochrane Central Registry of Controlled Trials from the earliest record to 19 March 2020. Only studies published in the English language were considered. The following terms were used both as a medical subject heading (MeSH) and free-text terms: “Roux-en-Y gastric bypass,” “sleeve gastrectomy,” “bariatric surgery,” “probiotics,” and “synbiotics.” Reference lists of each selected study were also searched by hand.

### 2.3. Data Extraction and Quality Assessment

The studies were selected by two independent researchers based on eligibility criteria. Extracted data involved author, publication year, country, study design, information of participants, intervention, and outcomes. The methodological quality within individual studies was also independently assessed by two researchers, via the Cochrane Collaboration's tool for judging the risk of bias in randomized trials [20]. Any disagreement was determined by discussion.

### 2.4. Statistical Analysis

This meta-analysis was accomplished via Review Manager (Version 5.3. Copenhagen: The Nordic Cochrane Centre, The Cochrane Collaboration, 2014). We pooled these data based on the duration of postoperational (less than 3 months or 12 months). All the outcomes in our meta-analysis were continuous variables, and standard mean differences (SMDs) with 95% confidence intervals (CIs) were calculated using the mean changes (standard deviations) from baselines to estimate the pooled effects. If not provided explicitly, we calculated the missing standard deviations (SD) of the changes using the following formula suggested by the Cochrane Handbook [21]: SD2 = SD baseline 2 + SD final 2-(2 × Corr × SD baseline × SD final). The correlation coefficient (Corr) was calculated by using sufficient data reported in the included studies and finally Corr = 0.6. The pooled effect *p* < 0.05 was regarded as statistically significant. The data unsuitable for quantitative analysis was limited to assess descriptively. We pooled these data based on the duration of post-operation (less than 3 months or 12 months).

The Chi2 test assessed heterogeneity, and the extent of heterogeneity was evaluated by the *I*^2^ test. Chi2 test with *p* < 0.10 and *I*^2^ > 50% indicated a significant degree of heterogeneity, and a random-effects model was used for analysis; otherwise, a fixed-effects model was applied. Publication bias was evaluated by the funnel plot, along with statistical estimates from Egger's test.

## 3. Results

### 3.1. Description of Included Studies

**Figure 1:**
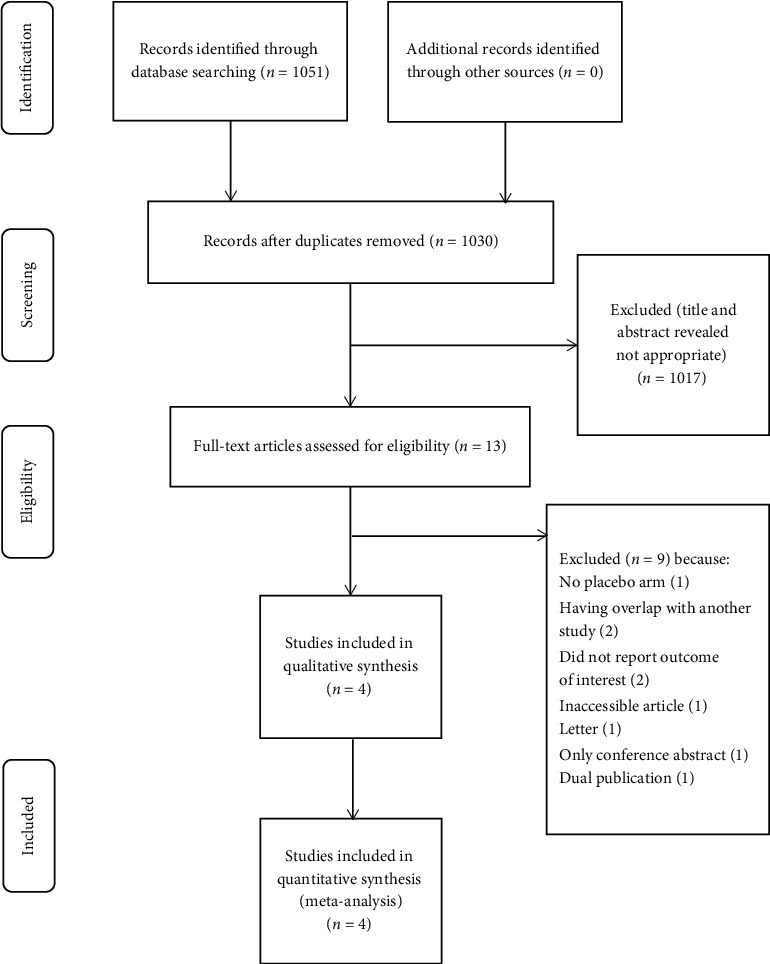
PRISMA flowchart of the process for searching and selecting studies.

We used a PRISMA flowchart to present the process of study selection for this meta-analysis. A total of 1051 records were identified in the initial electronic search based on the eligibility criteria, and finally, only four randomized controlled trials [22–25], including 172 participants, met the selection criteria (Figure 1).

Table 1 presents the characteristics of these four studies. Weight, BMI, %EWL, and WC were included as the primary outcomes. C-reactive protein (CRP), as one of the inflammatory factors with a significant positive correlation with obesity, was also included [26, 27]. We counted adverse events during these studies as well.

### 3.2. Risk of Bias

**Figure 2:**
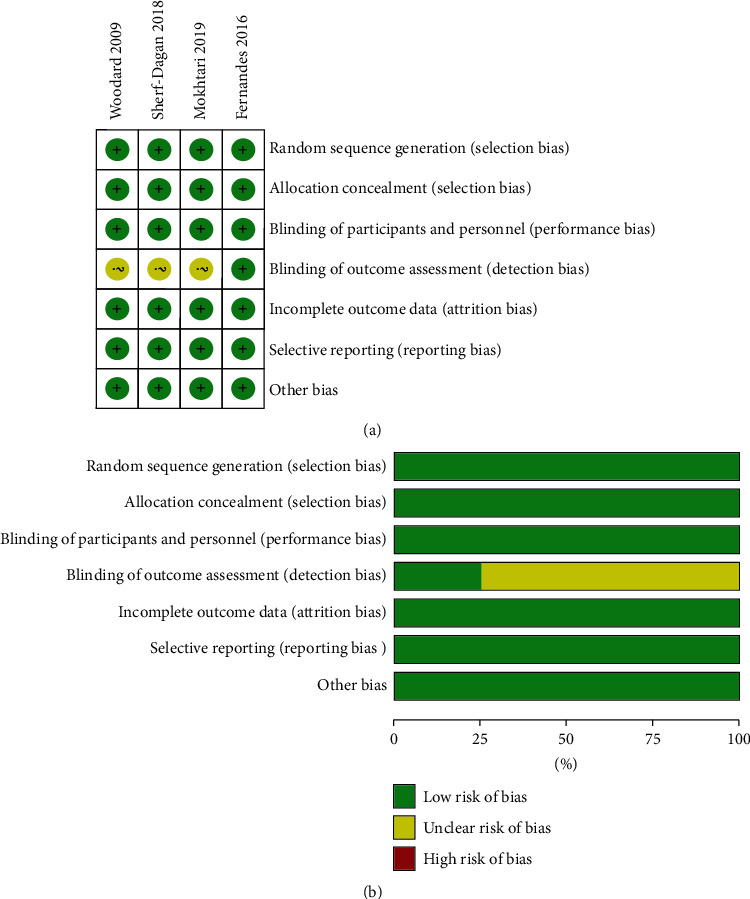
(a) Risk of bias summary: judgements about each risk of bias item for included studies. The green circle represents a low risk, and yellow represents an unclear risk, which means that no evidence was found. (b) Risk of bias graph: judgements about each risk of bias item presented as percentages across all included studies. The risk of bias is relatively low.

Figure 2(a) summarizes, as a table of judgements, each risk of bias item for included studies. Judgements about the risk of bias items across all are shown in Figure 2(b). Four trials had adequately described the method of randomization and assessed as quite a low risk of bias.

### 3.3. Meta-Analysis Outcomes

#### 3.3.1. Weight

**Figure 3:**
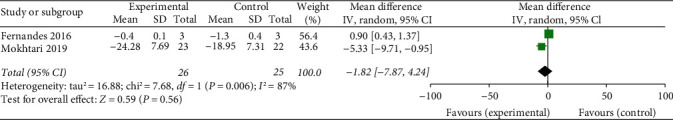
Forest plot presenting the effects of probiotic supplementation on the changes of weight in patients at 3 months after bariatric surgery.

Among the 4 RCTs examined, two [22, 23] were included in the meta-analysis of the effect of probiotics on body weight within 3 months after bariatric surgery. One [22] showed a significant decrease in the probiotic arm, while the other [23] showed no change between two arms. Then, a meta-analysis pooling revealed no significant differences between the probiotics treatment group and the control group (*p*=0.56), with a considerable heterogeneity between the studies (*p*=0.006, *I*^2^ = 87%) (Figure 3).

#### 3.3.2. Body Mass Index (BMI)

**Figure 4:**
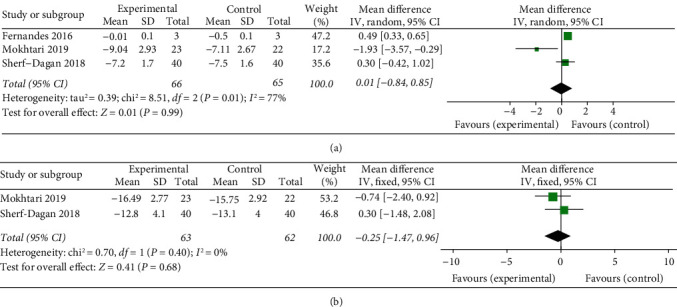
Forest plot presenting the effects of probiotic supplementation on the changes of BMI in patients at (a) 3 months and (b) 12 months after bariatric surgery, respectively.

Three [22, 23, 25] of included trials provided outcomes of BMI, while two [22, 25] had postoperative 12-month follow-ups. Fernandes et al. [23] found that the placebo group declined significantly compared with the synbiotic group at 15 days after BS. Mokhtari et al. [22] showed the advantage of the probiotic group in 3-month follow-up, but this advantage was not significant at 12 months. The outcomes in the study of Sherf-Dagan et al. [25] were always without statistical difference. When the data were pooled for meta-analysis, there was no significant reduction in BMI following probiotic supplementation within 3-month follow-up (*p*=0.99) with a huge heterogeneity (*p*=0.01, *I*^2^ = 77%) (Figure 4(a)), and at 12-month follow-up (*p*=0.68) without heterogeneity (*p*=0.40, *I*^2^ = 0%) (Figure 4(b)), respectively.

#### 3.3.3. Percentage of the Excess Weight Loss (%EWL)

**Figure 5:**
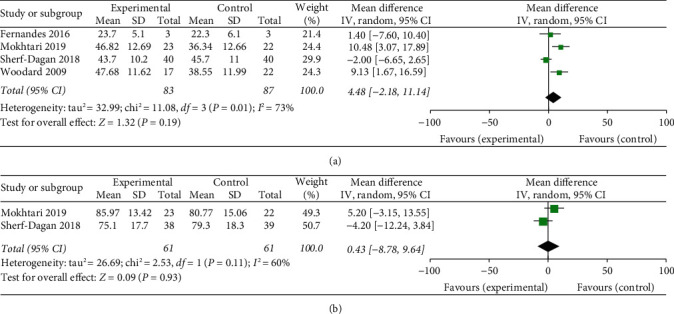
Forest plot presenting the effects of probiotic supplementation on the changes of %EWL in patients at (a) 3 months and (b) 12 months after bariatric surgery, respectively.

Among the four trials, two [22, 24] suggested statistically significant reductions in %EWL within 3-month follow-up. At 12 months, only two [22, 25] of all selected studies were included with no statistical significance. A meta-analysis of all four trials also indicated no significant differences in %EWL between the treatment group and the control group (*p*=0.19) throughout a 3-month follow-up, whereas the support of interstudy heterogeneity was observed (*p*=0.01, *I*^2^ = 73%) (Figure 5(a)). At 12-month follow-up, the meta-analysis was also not significant (*p*=0.93). The included studies were heterogeneous as well (*p*=0.11, *I*^2^ = 60%) (Figure 5(b)).

#### 3.3.4. Waist Circumference (WC)

**Figure 6:**
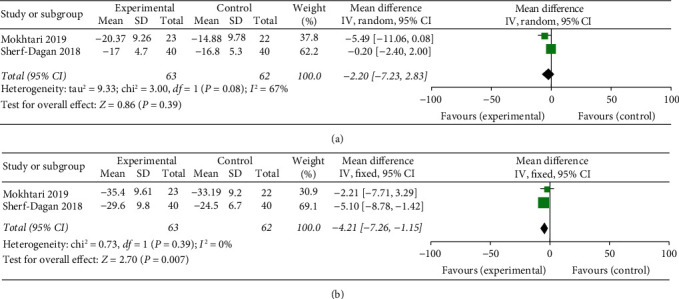
Forest plot presenting the effects of probiotic supplementation on the changes of WC in patients at (a) 3 months and (b) 12 months after bariatric surgery, respectively.

Two [22, 25] studies reported on the changes in WC, and there did not seem to be a significant difference in the probiotic groups compared with the placebo. Only one study [22] reported a marginal statistical significance in WC decline in the probiotic group at 3 months. The pooled data indicated no significant differences in WC at 3 months after surgery (*p*=0.39), with heterogeneity between the studies (*p*=0.08, *I*^2^ = 67%) (Figure 6(a)). However, at postoperative 12-month follow-up, the probiotic groups showed a statistically better effect on WC decline compared with the placebo (*p*=0.007), with the included studies homogeneous (*p*=0.39, *I*^2^ = 0%) (Figure 6(b)).

#### 3.3.5. C-Reactive Protein (CRP)

**Figure 7:**
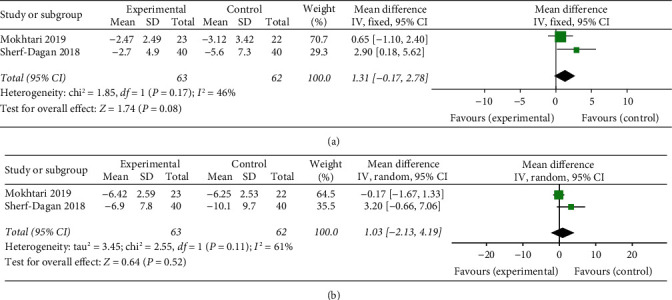
Forest plot presenting the effects of probiotic supplementation on the changes of CRP in patients at (a) 3 months and (b) 12 months after bariatric surgery, respectively.

Both included trials [22, 25] showed no statistically significant decrease in the probiotic group. The data pooled for analysis showed there was also no significant reduction in CRP between the probiotic and the placebo at 3-month follow-up (*p*=0.08) with a moderate heterogeneity (*p*=0.17, *I*^2^ = 46%) (Figure 7(a)), and at 12-month follow-up (*p*=0.52) with high heterogeneity (*p*=0.11, *I*^2^ = 61%) (Figure 7(b)), respectively.

### 3.4. Adverse Events

No studies reported severe adverse effects. One [23] reported that the patients developed excessive flatulence in the first 3 days of synbiotic supplementation, but no one dropped out of the study for this reason.

## 4. Discussion

This meta-analysis aimed to explore the adjunctive clinical effect of probiotics in obesity subjects after BS. We did not find meaningful evidence to suggest the benefit of probiotic supplements on weight, BMI, %EWL, and CRP within 3 months and at 12 months after BS. However, we found a statistically significant effect on WC in patients with BS at 12-month follow-up, instead of 3 months after BS.

Our meta-analysis showed only a significant reduction of −4.21 cm in WC for the probiotic group, instead of no effect on weight, %EWL, BMI, and CRP. Several articles provided similar results in studies of people with obesity. A meta-analysis by Suzumura et al. [28] pooled data from nineteen RCTs, including 1412 overweight and adults with obesity, and reported that probiotic or synbiotic supplementation reduced WC with a limited effect (MD = −0.82 cm), but no significant change in body weight or BMI. Similarly, a few RCTs demonstrated the positive impact of probiotics on WC in adults with obesity as well, but no significant effect on other indicators [13, 29]. Moreover, the results were also observed in children and adolescents. A randomized, triple-blind trial conducted in 64 obesity children found WC decreased in the probiotic group, without significant change in weight and BMI [30]. WC, not BMI, is a crucial measure of the severity of obesity and is more closely associated with obesity-related metabolic diseases [31, 32]. Here is a unique phenomenon-probiotics that appeared to be able to alter fat distribution without changing the body's total weight in subjects with obesity undergoing BS, which is reflected only in the decline in WC-meaning that the use of probiotics seems to be more beneficial to patients with obesity and related metabolic diseases. However, these four articles included in the analysis did not collect the effects of probiotics on the relief of obesity-related metabolic diseases in patients with obesity after BS, and more RCTs are needed.

Some underlying mechanisms might explain the decline in WC. Certain probiotics are used to assist in the improvement of gut microbiota [33] and are involved in inhibiting the expression of adipogenesis and adipocyte differentiation-related genes in the host [34–36]. Recent studies suggest that probiotics regulate the expression of adipogenesis genes in the liver to reduce visceral adipose tissue [37, 38]. The decrease in abdominal fat accumulation is manifested as a decrease in WC. Otherwise, fecal short-chain fatty acids (SCFAs) were observed to increase after probiotic supplement [39]. SCFAs bind with *G* protein-coupled receptors and affect the expression of intestinal hormones, which act with various insulin-sensitive tissues, which consequently leads to an improvement in obesity and a reduction in WC [40, 41].

We found no significant effect of probiotics on weight, BMI, %EWL, and CRP in obesity undergoing BS. Possibly, BS has a massive impact on obesity and metabolism; hence, the minor role of probiotics is hidden. Meanwhile, some meta-analyses have also found the unavailability of probiotics in subjects with obesity [42–44]. Here are what we infer: (1) subjects essential characteristics. Overweight but not subjects with obesity were more likely to benefit from probiotic supplements [45, 46] because excessive fat accumulation is associated with more inferior metabolic disorders [47, 48]. The subjects of our included trials were morbidly obese, and may be it caused negative results. (2) Different gut microbial enterotype. Compared with the *Bacteroides*-dominant enterotype, the *Prevotella*-dominant enterotype benefited more from probiotic supplements in the reduction of obesity-related markers, such as fat area and waist circumference [49]. (3) Type, dose, and duration of probiotic supplement. The probiotics consisting of multiple strains have significant effects, rather than a single strain [50]. High-dose probiotics appear to be more likely to affect gut microbiota and the host [13]. The longer intervention duration is associated with weight loss, inferring from several other meta-analyses [51, 52].

There are several strengths in this review. It is the first meta-analysis study on RCTs to explore the value of probiotics in the population with obesity undergoing BS. Besides, we calculated the correlation coefficients for each group according to the guidelines in Cochrane's handbook, and change from baseline was selected as a practical value to reduce variability in subjects' status. Finally, we planned to group by follow-up time to minimize heterogeneity.

There are several limitations in this review. Although these 4 studies included were RCTs, we cannot make a decision for sure due to the too small sample size (*n* = 172). Shorter follow-up data were included (3 and 12 months after BS), which may have resulted in less significant results. In addition, we pooled data from up to three months for analysis, which further affected the results. Our review only covered patients with morbid obesity, and the conclusions cannot be directly extended to people with different levels of obesity. We did not assess subgroup analysis as well as publication bias due to the only four trials and the limited data included. Together, we need to evaluate the results carefully.

These four experiments had short follow-up time. Our meta-analysis found no change in WC at 3 months after probiotics use and a significant decrease at 12 months. This also suggests that we need to extend the follow-up time. During the four studies, the participants were not operated on the same BS. The structure of the gastrointestinal tract can be dramatically altered by different BS procedures, which may influence the interaction between probiotics and gut microbes. The type of participants was only morbid obesity, which may be a limitation for the use of probiotics in populations with obesity of varying degrees. Finally, the use of probiotics varied in these experiments.

Probiotics can assist BS to reduce WC in patients with morbid obesity, which optimizes the overall weight loss and metabolic outcomes. However, this review is only a preliminary exploration of probiotics in this field (assisting BS weight loss and improving metabolic disorders). Future clinical trials with larger samples and long-term interventions are needed to study the efficacy and safety. At the same time, probiotics specific to obesity and metabolic diseases should be further explored to determine whether they are commonly used in routine clinical use.

## 5. Conclusions

Probiotics aid adults with morbid obesity in achieving further waist circumference improvement after BS, but with no significant effect on weight, BMI, %EWL, and CRP. More quality clinical studies are needed to confirm the efficacy and safety of probiotics and address a number of practical issues before the routine clinical use of probiotics in adults with obesity undergoing BS.

## Figures and Tables

**Table 1:** Characteristics of studies included in the meta-analysis.

Author (year)	Country	Study design	Participants	Intervention/control, BMI (kg/m^2^)	Intervention	Analyzed outcomes included
Fernandes et al. [23]	Brazil	Parallel RCT, triple-blind	Total 6 obese patients undergoing RYGB (mean age intervention 36.7 years, control 32.0 years)	40.2/38.2	100 ml water containing 6 of synbiotics^*∗*^ for 15 days	BMI, %EWL
Mokhtari et al. [22]	Iran	Parallel RCT, double-blind	Total 45 obese patients undergoing OAGB-MGB (mean age intervention 32.35 years, control 36.95 years)	44.59/44.95	Each capsule containing 7 species of probiotics^*∗∗*^ for 4 months	BMI, %EWL, WC, CRP
Sherf-dagan et al. [25]	Israel	Parallel RCT, double-blind	Total 80 obese patients undergoing LSG (mean age intervention 42.1 years, control 44.2 years)	42.1/42.1	Each capsule containing at least 25 billion active bacteria (11 species)^*∗∗∗*^ for 6 months	BMI, %EWL, WC, CRP
Woodard et al. [24]	USA	Parallel RCT, double-blind	Total 41 obese patients undergoing RYGB (mean age intervention 48.6 years, control 41.2 years)	45.7/49.6	Each probiotic capsule containing 2.4 billion live cells of *Lactobacillus* species for 6 months^*∗∗∗∗*^	%EWL

RCT: randomized controlled trial; RYGB: Roux-en-Y gastric bypass; OAGB-MGB: one-anastomosis gastric bypass; LSG: sleeve gastrectomy; FOS: fructo-oligosaccharide; BMI: body mass index; %EWL: percentage of the excess weight loss; WC: waist circumference; CRP: C-reactive protein; ^*∗*^synbiotics: FOS + 1 × 10^9^*Lactobacillus paracasei* LPC-37, 1 × 10^9^*Lactobacillus rhamnosus* HN001, 1 × 10^9^*Lactobacillus acidophilus* NCFM, and 1 × 10^9^*Bifidobacterium lactis* HN019; placebo: maltodextrin; ^*∗∗*^7 species of probiotics: *Lactobacillus casei* (3.5 × 109 CFU/g), *Lactobacillus rhamnosus* (7.5 × 108 CFU/g), *Streptococcus thermophiles* (1 × 108 CFU/g), *Bifidobacterium breve* (1 × 1010 CFU/g), *Lactobacillus acidophilus* (1 × 109 CFU/g), *Bifidobacterium longum* (3.5 × 109 CFU/g), and *Lactobacillus bulgaricus* (1 × 108 CFU/g) and 38.5-mg fructo-oligosaccharide; placebo: maltodextrin; ^*∗∗∗*^11 species of probiotics: *Lactobacillus acidophilus, Bifidobacterium bifidum, Lactobacillus rhamnosus, Lactococcus lactis, Lactobacillus casei, Bifidobacterium breve, Streptococcus thermophiles, Bifidobacterium longum, Lactobacillus paracasei, Lactobacillus plantarum,* and *Bifidobacterium infantis*; placebo: not provided; ^*∗∗∗∗*^placebo: not provided.

## Data Availability

The data supporting the results of this study are available within the article.
